# Gas and Steam Permeation Properties of Cation-Exchanged ZSM-5 Membrane

**DOI:** 10.3390/membranes15030070

**Published:** 2025-03-01

**Authors:** Yuichiro Hirota, Masaki Nakai, Kasumi Tani, Koya Sakane, Ayumi Ikeda, Yasuhisa Hasegawa, Sadao Araki

**Affiliations:** 1 Department of Life Science and Applied Chemistry, Nagoya Institute of Technology, Gokiso-cho, Showa-ku, Nagoya 466-8555, Japan; 2 National Institute of Advanced Industrial Science and Technology (AIST), Research Institute for Chemical Process Technology, 4-2-1 Nigatake, Miyagino-ku, Sendai 983-8551, Japan; a-ikeda@aist.go.jp (A.I.); yasuhisa-hasegawa@aist.go.jp (Y.H.); 3 Department of Chemical Engineering, Kansai University, 3-35 Yamatecho 3-Chome, Suita 564-8680, Japan; araki_sa@kansai-u.ac.jp

**Keywords:** ZSM-5 membrane, cation exchange, dehydration, H_2_O/H_2_ separation

## Abstract

NaZSM-5 powder and membranes were hydrothermally prepared. Their (1) steam (H_2_O) adsorption properties and (2) the permeation and separation of gas and H_2_O were evaluated before and after the cation exchange of Na^+^ to K^+^ or Cs^+^. The quantity of adsorbed H_2_O decreased as the size of the cation increased, indicating that the micropore volume and effective pore size of ZSM-5 decreased after cation exchange. The H_2_ and N_2_ permeances after cation exchange were less than 5% of the values before cation exchange, indicating a significant reduction in gas permeability. In contrast, the reduction of the H_2_O permeance values of the ZSM-5 membranes before and after K^+^ or Cs^+^ exchange was lower than that of H_2_, resulting in improved H_2_O/H_2_ separation performance. Compared with the NaZSM-5 membrane, the K^+^- or Cs^+^-exchanged ZSM-5 membranes exhibited superior H_2_O permselectivity, particularly at dilute H_2_O concentrations (<1 vol%).

## 1. Introduction

CO_2_ utilization technology is essential for achieving carbon neutrality and a circular economy. The conversion of CO_2_ and H_2_ to (1) methanol, (2) CO, via the reverse water gas shift (RWGS) reaction, and (3) fuel, via the Fischer–Tropsch reaction, can be considered as CO_2_ utilization technologies. However, challenges, such as thermodynamic constraints and/or slow reaction rates, limit these reactions. Membrane reactors can be used for the process intensification of these reactions. The selective removal of H_2_O from the reaction system via dehydration membranes can improve their CO_2_ conversion. Thus far, various H_2_O/H_2_ separation membranes, such as Nafion [1], silicon/rubber/ceramic composites [2], zeolite [3,4,5,6,7,8,9], organosilica [10,11,12], and ionic liquid-modified metal organic framework membranes [13], have been developed. Nafion [1], silicon rubber/ceramic composites [2], and zeolite membranes [14,15] have also been used in membrane reactor systems. Sakai et al. assessed the configuration of an RWGS membrane reactor using ZSM-5 membranes [15]. Although the membrane reactor exhibited a higher CO yield than that of the conventional reactor, the CO yield was further increased in the reactor composed of a combination of the conventional and membrane reactors. This was observed because the RWGS reaction proceeded in the conventional reactor in the first stage, and a mixture containing H_2_O of equilibrium composition was supplied to the ZSM-5 membrane, which was highly effective in inhibiting H_2_ and CO_2_ permeation through selective adsorption and capillary condensation of H_2_O in the micropores. Therefore, the H_2_O concentration dependence of H_2_O permselectivity in the design of membrane reactors is important. The kinetic diameters of H_2_O and H_2_ are 0.2955 nm [16,17] and 0.289 nm [18], respectively. Therefore, separation of H_2_O from H_2_ by molecular sieving is not possible. Selective adsorption or absorption of H_2_O is the main mechanism of H_2_O/H_2_ separation. However, performance degradation is inevitable at high temperatures and/or under dilute H_2_O conditions because of the adsorption and dissolution mechanisms.

In this study, cation exchange was used to improve the H_2_O permselectivity of ZSM-5 membranes under dilute H_2_O conditions. Aluminosilicate and silicoaluminophosphate zeolites are known to exhibit cation exchange capacity; additionally, the pore size and adsorption properties of zeolites are affected by the cations. For example, LTA-type zeolites containing K^+^, Na^+^, and Ca^2+^ can be used as 3A, 4A, and 5A molecular sieves, respectively. Low-silica X-type zeolites with Li^+^ ions are used during O_2_ pressure swing adsorption processes. In the field of membrane separation, the gas permeation and pervaporation properties of cation-exchanged ZSM-5 [19], FAU [20,21,22,23,24], Beta [25], and SAPO-34 [26] membranes have been reported. Based on the balance in changes in adsorption and diffusion, zeolite membranes showed different permeation and separation performance after cation exchange. The CO_2_/N_2_ permeation selectivity of a NaY zeolite membrane increased from 19 to 34–40 following exchange with K^+^, Rb^+^, and Cs^+^ because of the increase of adsorption selectivity [21]. Silver ion-exchanged FAU and Beta membranes are used for the separation of C_3_H_6_/C_3_H_8_ and C_2_H_4_/C_2_H_6_ [22,23,25]. Alkene permeation and separation performance through the membranes were improved after the Ag^+^ exchange due to strong interaction between Ag^+^ and alkenes. Ideal and separation selectivities of H-SAPO-34 membranes for H_2_/CH_4_ and CO_2_/CH_4_ increased after the cation exchange [26]. However, gas permeability decreased after cation exchange with Li^+^, Na^+^, K^+^, NH_4_^+^, and Cu^2+^, and the decrease was larger for large cations. Focusing on separation of H_2_O and H_2_ through the ZSM-5 membrane, only the differences between Na and H ions have been only discussed [7]. Steam permselectivity decreased after cation exchange from Na^+^ to H^+^. To the best of our knowledge, improvement of H_2_O permselectivity of ZSM-5 membranes by cation exchange has not been reported.

NaZSM-5 zeolite powder and membranes were synthesized in this study; thereafter, the Na^+^ ions were replaced by K^+^ and Cs^+^. First, Ar adsorption measurements were performed to assess the effect of cations on microporosity using powder samples. The quantity of adsorbed H_2_O and the adsorption enthalpy before and after cation exchange were also evaluated via H_2_O adsorption measurements using powder samples to discuss the effect of cation on H_2_O adsorption properties and microporosity. Second, (1) the effective pore size and (2) the permeation and separation properties of the ZSM-5 zeolite membranes before and after cation exchange were investigated using unary gas permeation and binary H_2_O/H_2_ separation tests at 473 K.

## 2. Materials and Methods

### 2.1. Synthesis of the NaZSM-5 Powder and Membrane

The NaZSM-5 powder was hydrothermally synthesized without an organic structure-directing agent using a modified method from a previous study [27]. An acidic amino acid addition technique [28,29] was used to prepare silicalite-1 nanocrystals, which were used as seed crystals. The precursor solution for the NaZSM-5 powder was prepared using colloidal silica (ST-S, Nissan Chemical Ind., Ltd., Yokohama, Japan), aluminum nitrate nonahydrate (FUJIFILM Wako Pure Chemical Industries Co., Osaka, Japan), sodium hydroxide (8 mol/L; FUJIFILM Wako Pure Chemical Industries Co., Osaka, Japan), and distilled water. The equivalent molar ratio was 1.0:0.02:0.125:27 (SiO_2_:Al_2_O_3_:Na_2_O:H_2_O). The precursor solution was then stirred at 353 K for 24 h. Thereafter, a silicalite-1 seed crystal containing 5 wt% SiO_2_ was added, then the precursor solution was stirred for 1 min at 298 K. Subsequently, the solution was transferred to a Teflon-lined autoclave (50 mL). Crystallization was performed for 24 h at 453 K under statistical conditions. The resulting powder was washed with distilled water and separated using centrifugation. The washing process was repeated in triplicate, and the samples were dried at 343 K for over 12 h.

NaZSM-5 membranes were prepared on the outer surface of a porous support using a secondary growth method. A porous hollow *α*-Al_2_O_3_ tube (outer diameter = 2 mm; inner diameter = 1 mm, length = 400 mm; average pore size = 150 nm) was used as the support. According to a previous study [30], the synthesis solution was prepared by mixing aluminum sulfate-(14-18)-hydrate (FUJIFILM Wako Pure Chemical Industries Co., Osaka, Japan), deionized water, sodium hydroxide (FUJIFILM Wako Pure Chemical Industries Co., Osaka, Japan), and colloidal silica (LUDOX HS-40; Sigma-Aldrich, Tokyo, Japan). This mixture was then stirred for 4 h at room temperature. The molar ratio of the synthesized solution was 220:1:60:30,000 (SiO_2_:Al_2_O_3_:Na_2_O:H_2_O). Silicalite-1 particles were dip-coated on the outside of the support tube, and both ends of the tube were sealed with Teflon tape to prevent crystal deposition on its inner surface. After removing the seals, the dip-coated support tube was dried at room temperature overnight and then calcined at 773 K for 1 h. Subsequently, the tube with both ends sealed was placed in a Teflon-lined stainless-steel autoclave containing the synthesis solution. The autoclave was then placed horizontally in an oven at 453 K for 20 h. After cooling to room temperature, the tube was removed from the autoclave and washed several times with deionized water. Synthesis of the NaZSM-5 zeolite membrane was completed after the tube was dried overnight at room temperature. The NaZSM-5 membrane (length = 400 mm) was cut into 30 mm pieces for the cation exchange treatment and permeation tests. The pieces were labeled NaZSM-5(1) and NaZSM-5(2), and cation exchanged with K^+^ and Cs^+^, respectively.

### 2.2. Cation Exchange

Potassium chloride and cesium acetate (FUJIFILM Wako Pure Chemical Industries Co., Osaka, Japan) were used as K and Cs salts, respectively. The NaZSM-5 powder was dispersed in a 1 mol/L K or Cs salt aqueous solution, then stirred at 343 K for 24 h. The resulting powder was washed with distilled water and separated via centrifugation. The washing process was repeated in sextuple, and the product was dried at 343 K for more than 12 h. Cation exchange of the NaZSM-5 membranes was also conducted using a similar procedure. The membrane was immersed in a 0.1 mol/L KCl or CH_3_COOCs aqueous solution and kept at 343 K for 1 h. The resulting membranes were washed with distilled water and dried at 383 K for more than 12 h. Hereafter, the cation-exchanged ZSM-5 samples will be referred to as KZSM-5 and CsZSM-5.

### 2.3. Characterization of ZSM-5 Powder and Membrane

X-ray diffraction (XRD) patterns (SmartLab SE using Cu Kα radiation; Rigaku, Tokyo, Japan) were used to identify the products. The morphologies of the ZSM-5 powders and membranes were examined using a field-emission scanning electron microscopy (FE-SEM; JSM-7001F, JEOL, Tokyo, Japan). Elemental analysis was conducted using energy-dispersive X-ray spectrometry (EDS) (X-Max^N^, Oxford Instruments, Abingdon-on-Thames, UK) for both the powder and membrane samples. The analysis for the membranes was performed on the ZSM-5 layer from the cross-sectional direction. It is noted that the SEM/EDS characterization on the NaZSM-5 membrane was performed using a different piece of the NaZSM-5(1) and NaZSM-5(2) after synthesis. For the KZSM-5 and CsZSM-5 membranes, the SEM/EDS characterization was conducted after the permeation tests described in Section 2.4. Argon adsorption measurements at 77 K were performed using a BELSORP-MAX II instrument (MicrotracBEL Co., Osaka Japan). Steam adsorption isotherms were measured for the powder samples at 303, 323, and 343 K using a BELSORP-aqua3 instrument (MicrotracBEL Co., Osaka, Japan). Prior to both of the adsorption tests, the powder samples were heated at 473 K for over 18 h under vacuum. The Langmuir equilibrium constant, *K* [kPa^−1^], was calculated from the isotherm across a low range of H_2_O pressure. Subsequently, the adsorption enthalpy, Δ*H* (kJ mol^−1^), was calculated from the temperature dependence of ln *K* using the van’t Hoff equation.

### 2.4. Permeation and Separation Tests

Unary gas permeation tests were conducted at 473 K to evaluate the effective pore sizes of the ZSM-5 membranes. Schematic of the gas permeation test apparatus is shown in Figure S1. Hydrogen, N_2_, and SF_6_ were used as the feed gases. Both ends of the membrane were connected to a stainless steel tube with a *poly*(1,1,2,2-tetrafluoroethylene)(PTFE)/fluorinated ethylene propylene (FEP) dual-layer heat-shrinkable tube (TOF S-130; SANPLATEC Co., Ltd., Osaka, Japan). The effective membrane area was 1.26 cm^2^. The membrane was installed in a custom-made permeation module, as described previously [31]. The feed gas stream was maintained at 200 kPa, and the permeated gases were collected by sweeping H_2_ or N_2_ gas. Table S1 shows gas flow rate in the unary gas permeation test. The permeated gases were then analyzed using a gas chromatograph equipped with a TCD detector (GC-8A, Shimadzu, Kyoto, Japan). The permeation performance of the membrane was evaluated using permeance (mol m^−2^s^−1^Pa^−1^).

During the binary H_2_O/H_2_ separation tests, the membranes were retained in the same membrane module used during the unary gas permeation tests. Hydrogen was passed through a saturator filled with distilled water at 298 K to feed the binary gas mixture into the membrane. The feed-side stream was maintained at 101 kPa. The partial pressures of H_2_O were adjusted from 0.4 to 3.2 kPa by diluting the gas mixture with H_2_. The permeation temperature increased from 373 to 473 K. The permeated components were collected by sweeping N_2_ gas. The permeated stream was analyzed using the GC-8A gas chromatograph. The separation performance of the membranes was evaluated using permselectivity, which was calculated as the permeance value ratio. Other details of the permeation and separation tests are described in the Supplementary Materials. 

## 3. Results and Discussions

### 3.1. Preparation of the ZSM-5 Powder and Membrane

**Figure 1:**
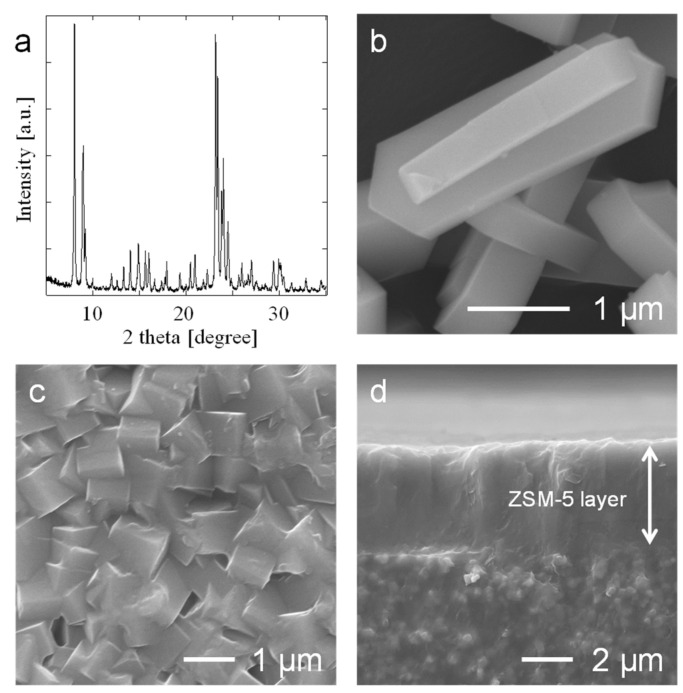
Characterization of the NaZSM-5 powder by (**a**) XRD measurements and (**b**) FE-SEM observation, and the as-made NaZSM-5 membrane by FE-SEM for (**c**) surface and (**d**) cross-section.

The XRD pattern and FE-SEM image of the NaZSM-5 powder are shown in Figure 1a,b. Diffraction peaks characteristic of the MFI structure were observed. The AlMFI powder contained crystalline particles approximately 3 × 1 μm in size, and their shape was coffin-like. Surface and cross-sectional FE-SEM images of the NaZSM-5 membrane are shown in Figure 1c,d. The membrane comprised a typical polycrystalline structure, and the thickness of the NaZSM-5 layer was approximately 4 μm.

The Si/Al ratios of both ZSM-5 powder and membrane before and after cation exchange are listed in Table 1. EDS spectra for the three membranes are shown in the Supplementary Materials. EDS analysis showed that the Si/Al ratios of the NaZSM-5 powder and membrane were 23 and 18, respectively. Therefore, NaZSM-5 powders and membranes with similar Al contents were successfully synthesized. In this study, both NaZSM-5 powder and membranes were synthesized without an organic structure direct agent, such as tetrapropylammonium salt. Only sodium hydroxide was used for synthesis. Therefore, the cation in the prepared samples is Na^+^. Figure S2 compares the XRD patterns of the ZSM-5 powder before and after the cation exchange. The cation exchange from Na^+^ to K^+^ and Cs^+^ had little influence on the framework structure of the parent NaZSM-5. Figure S3 shows cross-sectional FE-SEM images and EDS spectra of the ZSM-5 membranes. The Si/Al ratios were similar after the K^+^ and Cs^+^ exchange. The EDS analysis also showed that the molar values of Na/Al for both of the ZSM-5 powder and membranes after the cation exchange were less than 0.02. These results indicate that Na^+^ in the NaZSM-5 powder and membranes was successfully exchanged with K^+^ or Cs^+^.

### 3.2. Effect of Cations on Microporosity and H_2_O Adsorption

**Figure 2:**
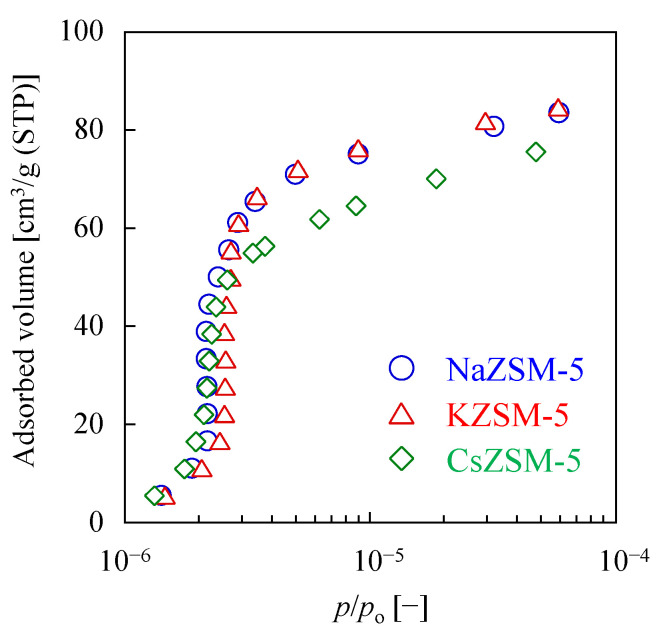
Ar adsorption isotherms at 77 K for NaZSM-5 (blue), KZSM-5 (red), and CsZSM-5 (green) powder.

Argon adsorption measurements were taken for the three types of ZSM-5 powder to discuss the effect of cation on micropore volume. Figure 2 shows the Ar adsorption isotherms for *p*/*p*_o_ values between 10^−6^ and 10^−4^. According to the Saito–Floy (SF) method, the value of 1 × 10^−5^ for *p*/*p*_o_ corresponds to a pore size of about 0.55 nm, which was the typical pore size for the MFI structure. The Ar adsorption capacity of the CsZSM-5 powder at *p*/*p*_o_ = 0.9 × 10^−5^ was 65 cm^3^/g (STP), which was about 85% of that of NaZSM-5. We confirmed a decrease of the micropore volume by exchanging the cation, from Na^+^ to larger Cs^+^. This result also suggests that the effective pore size would have decreased where the Cs^+^ is present. Although the ionic radius of K is larger than that of Na, a significant difference was not observed between the adsorption isotherms of the KZSM-5 and the NaZSM-5 powder. This would be due to (1) the small difference in size between K^+^ and Na^+^ compared to Cs^+^, and (2) the cation content of NaZSM-5. For these two reasons, the cation exchange to K^+^ would have made little effect on the micropore volume.

**Figure 3:**
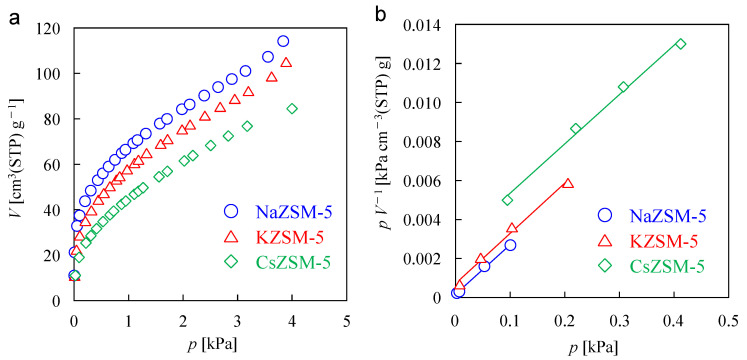
(**a**) Steam adsorption isotherm and (**b**) *p V*^−1^ vs. *p* plot at 303 K for the ZSM-5 powder samples.

Steam adsorption measurements were performed on three types of ZSM-5 powders to assess the cationic effect on Δ*H*, and the saturated adsorption quantity, V_s_. In this study, the Δ*H* values were not determined by direct measurements of heat changes during steam adsorption. Here, the objective of calculating Δ*H* values is to discuss the change in Δ*H* with cationic species. Figure 3a shows the steam adsorption isotherms at 303 K. The isotherms measured at 323 and 343 K are shown in Figure S4. A reduction in the quantity of adsorbed H_2_O at each temperature with increasing ionic radius (Na^+^ < K^+^ < Cs^+^) was observed, suggesting that the cations in ZSM-5 affect the *K* values.

Next, the cationic effects on *V*_s_ and Δ*H* were assessed. A linear relationship is expressed when a plot of *p V*^−1^ versus *p* is used for the adsorption isotherm. Using this expression, the isotherm can be analyzed according to the Langmuir adsorption model by using the following equation:

(1) $$V=V_{s}Kp{\left(1+Kp\right)}^{-1}$$

Equation (1) can be modified as follows:

(2) $$pV^{-1}=pV_{s}^{-1}+K^{-1}V_{s}^{-1}$$

where *V* (cm^3^(STP) g^−1^) is the quantity of adsorbed H_2_O at a specified pressure of *p* (kPa). *K* (kPa^−1^) and *V*_s_ (cm^3^(STP) g^−1^) are the Langmuir equilibrium constant and the saturated adsorption quantity, respectively. All isotherms exhibited linear relationships between *p V*^−1^ and *p* in the low range of H_2_O pressure, as shown in Figure 3b and Figure S5. Therefore, *V*_s_ and *K* can be calculated from the slope and intercept. Table 2 lists the calculated *V*_s_ values. With the exception of the value for NaZSM-5 at 343 K, similar values were obtained regardless of cation and measurement temperature. Different from the result of Ar adsorption measurements (Figure 2), the obtained results indicate that there is no significant difference in microporosity due to cations in the steam adsorption. The difference in (1) molecular size between H_2_O and Ar and (2) the cation content of ZSM-5 would have contributed to the difference in the effect of cations in the two adsorption measurements. The higher *V*_s_ value for the NaZSM-5 at 343 K would be due to the lower correlation between *p V*^−1^ and *p* than for the other two temperatures.

**Figure 4:**
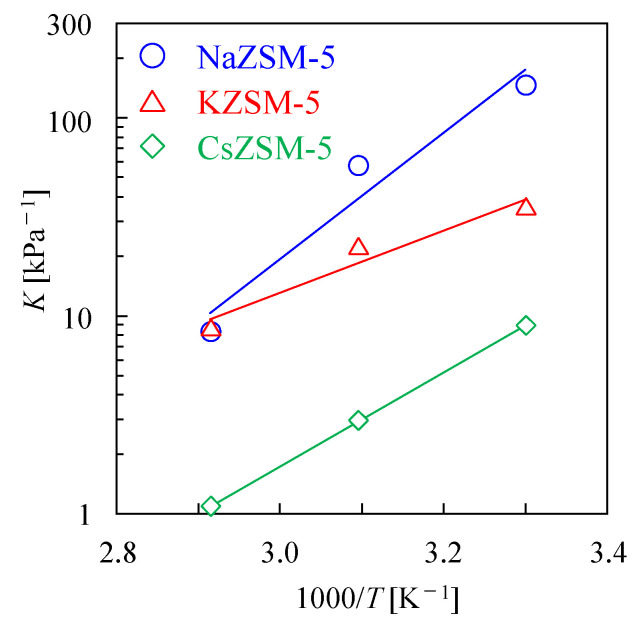
Temperature dependence of Langmuir equilibrium constant for the ZSM-5 powder samples.

The calculated *K* values are listed in Table S2, and their temperature dependence is shown in Figure 4.

The values of Δ*H* can be calculated from the temperature dependence of *K* using the following van’t Hoff equation:

(3) $$\frac{d\;ln\;K}{dT}=\frac{∆H}{RT^{2}}$$

Equation (3) can be modified as follows:

(4) $$\frac{d\;ln\;K}{d\left(1/T\right)}=\frac{∆H}{R}$$

where *R* (J K^−1^ mol^−1^) and *T* (K) are the gas constant and adsorption temperature, respectively. Therefore, the values of Δ*H* can be determined from the slope shown in Figure 4. The calculated values of Δ*H* from this and previous studies are listed in Table 3. In a previous study, Δ*H* values of −113 and −75 kJ mol^−1^ were determined for H^+^-type ZSM-5 (HZSM-5) with Si/Al ratios of 38 and 250, respectively, using thermogravimetric analyses [32]. Bolis et al. assessed the Δ*H* values of HZSM-5 (Si/Al ratios of 3.8) and silicate-1 using microcalorimetry. The values for HZSM-5 and silicate-1 ranged between −69 and −84 kJ mol^−1^ and between −61 and −68 kJ mol^−1^, respectively [33]. Ohlin et al. used attenuated total reflectance Fourier-transform infrared (ATR-FTIR) spectroscopy on NaZSM-5 films with a Si/Al ratio of 130 [34]. The Δ*H* values of the pore wall, and defects in the form of silanol groups and/or sites containing Na^+^ were −72 and −58 kJ mol^−1^, respectively. The NaZSM-5 powder used in this study exhibited a Si/Al ratio and Δ*H* value of 23 and −61 kJ mol^−1^, respectively. As mentioned above, the objective of the Δ*H* calculation is to discuss the change of Δ*H* values with cation. Although the Al content of NaZSM-5 and the measurement method differed from previous studies, the Δ*H* value was similar to those observed in previous studies.

The Δ*H* values increased after cation exchange, indicating a reduction in exothermic nature compared with that of NaZSM-5. Yang et al. assessed the hydration enthalpy of cation-exchanged Y-type zeolites. The hydration enthalpies (per mole of zeolite and per mole of H_2_O) for K^+^- and Cs^+^-exchanged Y-type zeolites were lower than those of the Na^+^-containing Y-type zeolites [35]. The difference in hydration enthalpies of zeolites with different cations was likely affected by several factors, including (1) ionic potential, (2) relation between cation size and micropore volume, (3) bonding power, and the (4) compatibility between the cation–water arrangement of the zeolite structure. These factors, as well as the Y-type zeolite, would also affect the ZSM-5 zeolite, resulting in a variation in the values of Δ*H* via cation exchange.

### 3.3. Effect on Gas Permeability and Effective Zeolitic Pore Size

**Figure 5:**
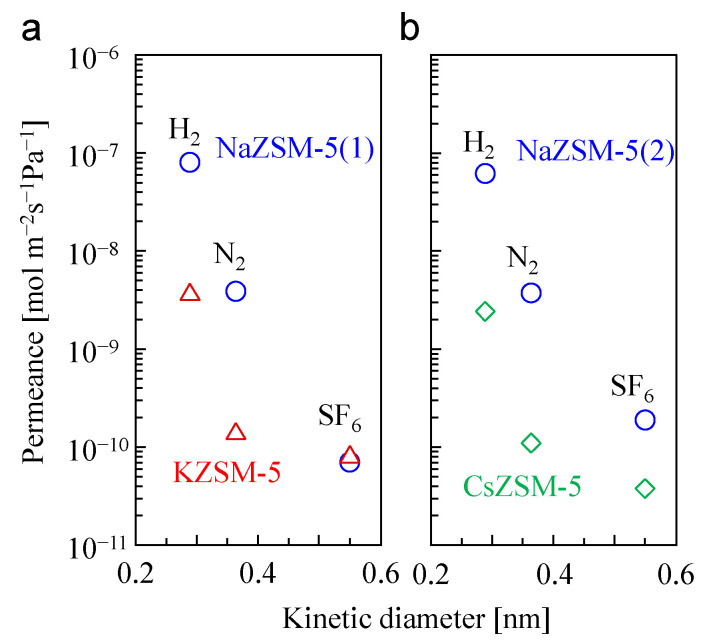
Unary gas permeation properties of ZSM-5 membranes before and after (**a**) K^+^ and (**b**) Cs^+^ exchange. Permeation temperature was 473 K.

Figure 5 and Table 4 present the results of the unary gas permeation tests. The gas permeances of the NaZSM-5 membranes decreased with increasing kinetic diameter. The ideal permselectivities of H_2_/SF_6_ and N_2_/SF_6_ for the two NaZSM-5 membranes were greater than 300 and 20, respectively. The separation performance for H_2_/SF_6_ is compared with those of other MFI-type zeolite membranes in Figure S6. Although the H_2_/SF_6_ permeance ratio tends to decrease as the permeation temperature increases, the synthesized NaZSM-5 membranes in this study showed high permselectivity of H_2_/SF_6_ at 473 K. This high H_2_/SF_6_ separation performance was attributed to the molecular sieving properties of the NaZSM-5 membrane with few defects. After cation exchange with K^+^ and Cs^+^, the gas permeances, particularly the H_2_ and N_2_ permeances, decreased significantly.

The permeance values of H_2_ and N_2_ via the KZSM-5 membrane were approximately 4.8 and 2.9% of those of NaZSM-5 membrane (1), respectively. The H_2_ and N_2_ permeances of the CsZSM5 membrane were approximately 3.9 and 3.7% of those of the NaZSM-5 membrane (2), respectively. The ideal permselectivities for N_2_/SF_6_ via KZSM-5 and CsZSM-5 were 2 and 3, respectively, indicating that neither membrane exhibited molecular sieving properties for the two gases. In contrast, the permselectivity for H_2_/N_2_ marginally increased after cation exchange. These results indicate that cation exchange from Na^+^ to K^+^ and Cs^+^ resulted in a reduction in the micropore volume of the ZSM-5 membrane; additionally, the effective zeolitic pore size was reduced to approximately 0.36 nm, which is similar to the kinetic diameter of N_2_. Kinetic diameters of H_2_ and Ar are close to those of H_2_O and N_2_, respectively. Compared to the results of Ar and steam adsorption measurements (Figure 2 and Table 2), the effect of cation exchange on micropore volume and pore size was greater on the membranes than on the powders. This behavior was related to the morphology of the membrane. The synthesized ZSM-5 membrane had a polycrystalline structure (Figure 1c), which results in a disconnection of the micropores at the grain boundary, leading to a reduction in the micropore volume and effective zeolitic pore size [36]. The unique structure of the ZSM-5 membrane resulted in a cation exchange effect, which differed from that observed in the Ar and steam adsorption measurements using the ZSM-5 powder.

### 3.4. Effect on H_2_O/H_2_ Permeation and Separation Performance

**Figure 6:**
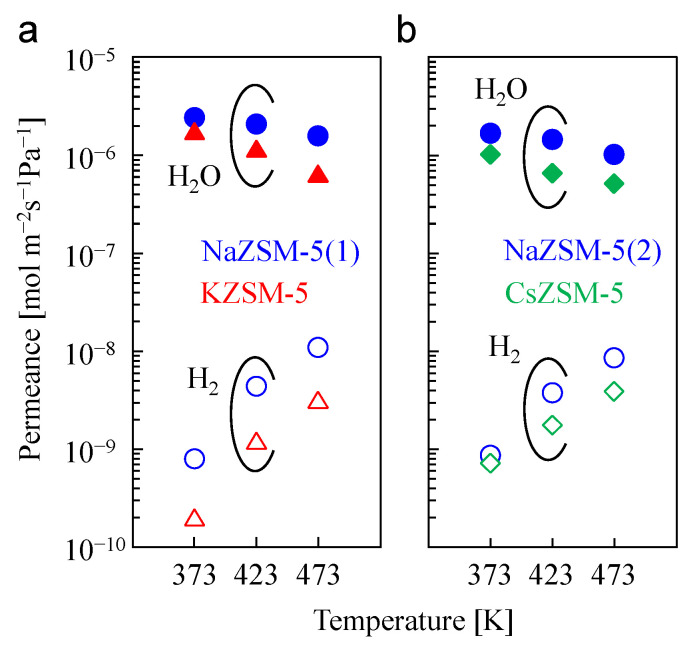
Temperature dependance of H_2_O and H_2_ permeances in the binary mixture system before and after (**a**) K^+^ and (**b**) Cs^+^ exchange. Partial pressure of H_2_O was 3.2 kPa. Close symbol, H_2_O; open symbol, H_2_.

Figure 6 shows the temperature dependence of H_2_O and H_2_ permeances in the binary mixture system. The separation tests were performed at an H_2_O partial pressure of 3.2 kPa. Although the H_2_O permeance decreased after cation exchange, the KZSM-5 and CsZSM-5 membranes exhibited selective permeation of H_2_O over H_2_ at temperatures ranging from 373 to 473 K, similar to the NaZSM-5 membranes.

The activation energy of H_2_O permeation (*E*_p,H2O_) through the membranes was calculated using the Arrhenius equation, and the resulting values are listed in Table 5. The Δ*H* values calculated with the H_2_O adsorption test using powder samples are listed again in Table 5. The *E*_p,H2O_ value for the KZSM-5 membrane (−14 kJ/mol) was lower than that of the NaZSM-5 membrane (−6.1 kJ/mol). Additionally, the *E*_p,H2O_ value after Cs exchange decreased from −7.3 kJ/mol to −10 kJ/mol.

In these experiments, the permeate side was swept with N_2_ of 300 to 400 cm (STP) min^−1^, and the H_2_O concentration on the permeation side was less than 0.3%; thus, desorption from the membranes on the permeation side would be negligible. Therefore, *E*_p,H2O_ can be expressed as the sum of Δ*H* and the activation energies of H_2_O diffusion inside the membrane, *E*_diffusion_, using Equation (5).

(5) $$E_{p,H2O}=∆H+E_{diffusion}$$

The calculated *E*_p,H2O_ values for each membrane indicated that the contribution of the H_2_O adsorption step to H_2_O permeation via the KZSM-5 and CsZSM-5 membranes was higher than that through the NaZSM-5 membranes. However, the results of the adsorption tests indicated that the values of Δ*H* for KZSM-5 and CsZSM-5 were higher than that for NaZSM-5, which would contribute to an increase in the *E*_p,H2O_ values. The reduction in the *E*_p,H2O_ values after cation exchange likely occurred because the *E*_diffusion_ value of the NaZSM-5 membrane was higher than those of the KZSM-5 and CsZSM-5 membranes owing to the strong H_2_O adsorption capacity of NaZSM-5. Because of its low Δ*H* value (=the strong H_2_O adsorption capacity), the desorption of the adsorbed H_2_O molecules inside the NaZSM-5 zeolitic pores was challenging, resulting in reduced diffusivity, even though its effective pore size was larger than those of KZSM-5 and CsZSM-5. Although the values of Δ*H* increased after K^+^ and Cs^+^ exchange, the reduction in the *E*_diffusion_ values would be greater than the increase of Δ*H* values. This would result in lower *E*_p,H2O_ values for the KZSM-5 and CsZSM-5 membranes.

**Figure 7:**
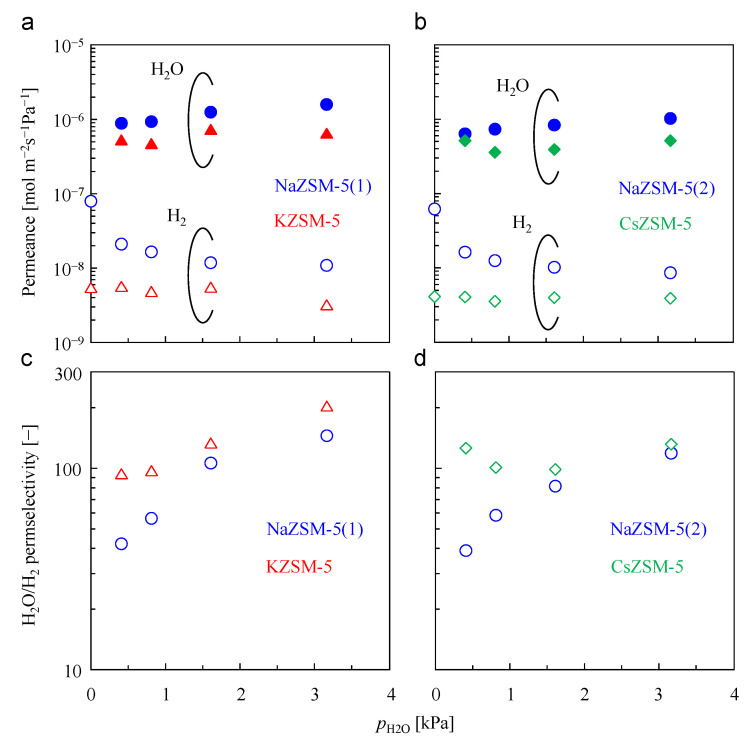
Permeation and separation properties of the membrane before and after (**a**,**c**) K^+^ and (**b**,**d**) Cs^+^ exchange as a function of partial pressure of H_2_O in a H_2_O/H_2_ binary mixture separation test. The total pressure of the feed side is 101 kPa, and permeation temperature is 473 K. Close symbol, H_2_O; open symbol, H_2_ (**a**,**b**).

Next, the effect of the H_2_O partial pressure in the feed stream on the permeation and separation performances of the ZSM-5 membranes before and after cation exchange was evaluated (Figure 7). For the NaZSM-5 membranes, H_2_O permeance slightly increased with increasing H_2_O partial pressure. However, the H_2_ permeance decreased with increasing H_2_O partial pressure, resulting in an increase in the H_2_O/H_2_ permselectivity. An increase in the H_2_O partial pressure increased the quantity of H_2_O adsorbed onto the membranes, which facilitated H_2_O permeation and inhibition of H_2_ permeation.

After cation exchange, the reduction in H_2_O and H_2_ permeances was similar to that observed in the unary permeation tests. However, the reduction in the H_2_O permeance values of the ZSM-5 membranes before and after K^+^ or Cs^+^ exchange was lower than that of H_2_ in the binary H_2_O/H_2_ separation tests, resulting in improved H_2_O/H_2_ separation performance. In particular, at ultra-dilute H_2_O concentrations (<1 vol%), H_2_O/H_2_ permselectivities greater than 90 were obtained for the KZSM-5 and CsZSM-5 membranes, which were significantly higher than those of the NaZSM-5 membranes (40–60). A significant improvement in the separation performance was observed. The presence or absence of H_2_O did not affect H_2_ permeance through KZSM-5 and CsZSM-5. This suggests that H_2_ permeation through both membranes was not inhibited by selective H_2_O adsorption onto the micropores. As displayed in Figure S5, the synthesized NaZSM-5 membranes in this study had fewer defects compared to other ZSM-5 membranes. In addition, the results of adsorption measurements and unary gas permeation tests strongly indicate that the unique polycrystalline structure of the zeolite membrane leads to a change in effective zeolitic pore size via cation exchange that is different from that of the powder. Although these factors would contribute to show the H_2_O concentration dependence of H_2_ permeance through the cation-exchanged ZSM-5 membranes, it is difficult to discuss the detailed mechanism in this study. Further analysis is required.

**Figure 8:**
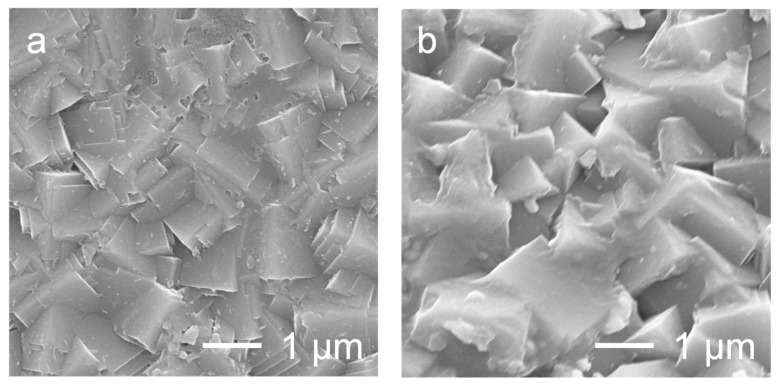
Surface FE-SEM images of the (**a**) KZSM-5 and (**b**) Cs-ZSM-5 membranes after the permeation tests.

Figure 8 shows surface FE-SEM images of the KZSM-5 and CsZSM-5 membranes after the permeation tests. Compared to the as-made NaZSM-5 membrane (Figure 1c), no significant changes were observed, suggesting that the KZSM-5 and CsZSM-5 membranes are expected to show high hydrothermal stability.

**Figure 9:**
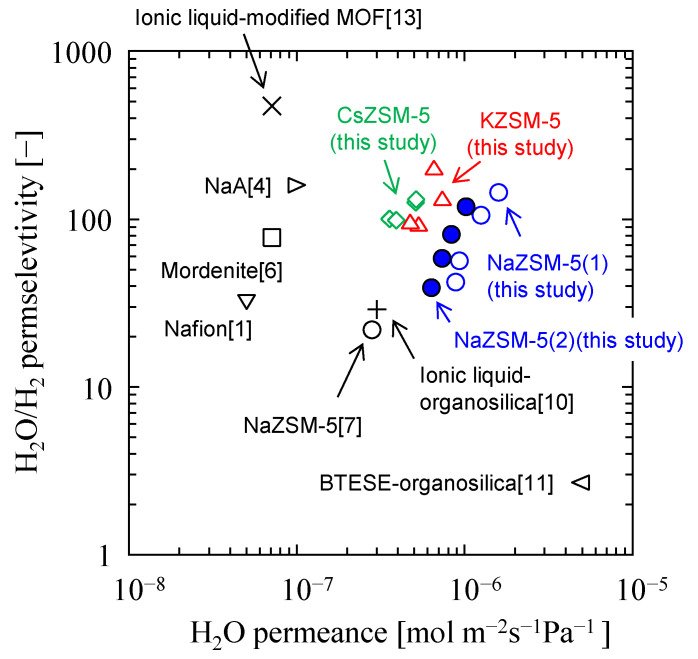
Relationships between H_2_O permeance and H_2_O/H_2_ permselectivity at temperature range from 473 to 523 K under H_2_O concentration less than 10 mol%. Close blue symbol, NaZSM-5(1); open blue symbol, NaZSM-5(2).

Finaly, the permeation and separation performance of the developed ZSM-5 membranes were compared with those of other membranes, as shown in Figure 9.

Steam permeances and H_2_O/H_2_ permselectvity of the NaZSM-5 membranes were higher than those of other membranes. The superior performance of the NaZSM-5 membranes can be attributed to their very few defects and support Al_2_O_3_ thickness. As mentioned above, H_2_O/H_2_ permselectivity increased after the cation exchange. Narrowing the effective zeolitic pore size via the cation exchange can be attributed to the enhancement of inhibition of H_2_ permeation. Cation exchange with K^+^ and Cs^+^ is a simple and effective approach for improving H_2_O permselectivity.

## 4. Conclusions

The NaZSM-5 powder and membranes were hydrothermally synthesized; thereafter, the Na^+^ ions were replaced by K^+^ and Cs^+^. A reduction in the micropore volume and effective pore size after cation exchange was confirmed using steam adsorption analyses. However, the reduction in permeance from the adsorption experiment was greater than expected. The unique polycrystalline structure of the zeolite membrane affected the obtained difference.

H_2_O and H_2_ permeances decreased after K^+^ and Cs^+^ exchange; however, the reduction in the H_2_O permeance values was lower than that of H_2_ from the binary H_2_O/H_2_ separation tests. The KZSM-5 and CsZSM-5 membranes exhibited higher H_2_O/H_2_ separation performances than that of the NaZSM-5 membrane. The contribution of the H_2_O adsorption step to H_2_O permeation via the KZSM-5 and CsZSM-5 membranes was higher than that of the NaZSM-5 membranes even though the H_2_O adsorption enthalpy increased via cation exchange. The weaker H_2_O adsorption properties of KZSM-5 and CsZSM-5 contributed to an improvement in H_2_O diffusivity inside the zeolitic pores. Therefore, lower activation energies for H_2_O permeation were obtained for the KZSM-5 and CsZSM-5 membranes.

The KZSM-5 and CsZSM-5 membranes exhibited H_2_O/H_2_ permselectivities greater than 90 at ultra-dilute H_2_O concentrations (<1 vol%), which were higher than those of the NaZSM-5 membranes (40–60). Cation exchange with K^+^ and Cs^+^ is a simple and effective approach for improving H_2_O permselectivity

## Figures and Tables

**Table 1:** Si/Al ratios of the ZSM-5 powder and membrane samples.

Sample		Si/Al
powder	NaZSM-5	23
	KZSM-5	22
	CsZSM-5	21
membrane	NaZSM-5	18
	KZSM-5	21
	CsZSM-5	17

**Table 2:** Saturated adsorption quantity for the ZSM-5 powder samples.

Temperature (K)	*V*_s_ (cm^3^(STP) g^−1^)		
	NaZSM-5	KZSM-5	CsZSM-5
303	39	39	40
323	42	43	42
343	48	42	42

**Table 3:** The adsorption enthalpy of H_2_O on ZSM-5 powder samples.

Zeolite	Si/Al	Δ*H* (kJ mol^−1^)	Ref
NaZSM-5	23	−61	this study
KZSM-5	22	−30	this study
CsZSM-5	21	−45	this study
HZSM-5	38	−113	[32]
HZSM-5	250	−75	[32]
HZSM-5	3.8	−69 to −84	[33]
silicalite-1	∞	−61 to −68	[33]
NaZSM-5	130	−72 (site 1) −58 (site 2)	[34]

**Table 4:** Ideal permselectivity of ZSM-5 membranes before and after cation exchange.

Membrane	Ideal Permselectivity *		
	H_2_/N_2_	H_2_/SF_6_	N_2_/SF_6_
NaZSM-5(1)	21	1141	55
KZSM-5	26	45	2
NaZSM-5(2)	17	329	20
CsZSM-5	22	64	3

* Calculated using the ratio of permeance values in the unary system.

**Table 5:** Comparison of *E*_p,H2O_ and Δ*H* values before and after cation exchange.

	*E*_p,H2O_ (kJ/mol)	Δ*H* (kJ mol^−1^) *
NaZSM-5	−6.1 (membrane(1)) −7.3 (membrane(2))	−61
KZSM-5	−14	−30
CsZSM-5	−10	−45

* Calculated from the data using powder samples.

## Data Availability

The raw data supporting the conclusions of this article will be made available by the authors on request.

## Supplementary Materials

The following supporting information can be downloaded at: https://www.mdpi.com/article/10.3390/membranes15030070/s1, Figure S1: Schematic of the gas permeation test apparatus; Table S1: Gas flow rate in the unary gas permeation test; Figure S2: XRD patterns of ZSM-5 powder samples; Figure S3: Cross-sectional FE-SEM images and EDS spectra of (a) NaZSM-5, (b) KZSM-5, and (c) CsZSM-5 membranes; Figure S4: Steam adsorption isotherm of the ZSM-5 powder samples at (a) 323 and (b) 343 K; Figure S5: p V^−1^ vs. p plot of the ZSM-5 powder samples at (a) 323 and (b) 343 K; Table S2: Langmuir constants for the ZSM-5 powder samples; Figure S6: Relationship of H2 permeance and H2/SF6 permeation ratio of MFI-type zeolite membranes. References [37,38,39,40,41,42,43,44,45,46,47,48,49,50,51,52] are cited in the Supplementary Materials.

## References

[1] Struis R.P.W.J., Stucki S., Wiedorn M. (1996). A membrane reactor for methanol synthesis. J. Membr. Sci..

[2] Chen G., Yuan Q. (2004). Methanol synthesis from CO_2_ using a silicone rubber/ceramic composite membrane reactor. Sep. Purif. Technol..

[3] Piera E., Salomón M.A., Coronas J., Menéndez M., Santamaría J. (1998). Synthesis, characterization and separation properties of a composite mordenite/ZSM-5/chabazite hydrophilic membrane. J. Membr. Sci..

[4] Aoki K., Kusakabe K., Morooka S. (2000). Separation of gases with an A-type zeolite membrane. Ind. Eng. Chem. Res..

[5] Sato K., Sugimoto K., Sekine Y., Takada M., Matsukata M., Nakane T. (2007). Application of FAU-type zeolite membranes to vapor/gasseparation under high pressure and high temperature up to 5 MPa and 180 °C. Micropor. Mesopor. Mater..

[6] Sawamura K., Shirai T., Ohsuna T., Hagino T., Takada M., Sekine Y., Kikuchi E., Matsukata M. (2008). Separation behavior of steam from hydrogen and methanol through mordenite membrane. J. Chem. Eng. Jpn..

[7] Sawamura K., Izumi T., Kawasaki K., Daikohara S., Ohsuna T., Takada M., Sekine Y., Kikuchi E., Matsukata M. (2009). Reverse-selective microporous membrane for gas separation. Chem. Asian J..

[8] Sandström L., Palomino M., Hedlund J. (2010). High flux zeolite X membranes. J. Membr. Sci..

[9] Wang H., Lin Y.S. (2012). Effects of water vapor on gas permeation and separation properties of MFI zeolite membranes at high temperatures. AIChE J..

[10] Hirota Y., Yamamoto Y., Nakai T., Hayami S., Nishiyama N. (2018). Application of silylated ionic liquid-derived organosilica membranes to simultaneous separation of methanol and H_2_O from H_2_ and CO_2_ at high temperature. J. Membr. Sci..

[11] Moriyama N., Nagasawa H., Kanezashi M., Tsuru T. (2019). Selective water vapor permeation from steam/non-condensable gas mixtures via organosilica membranes at moderate-to-high temperatures. J. Membr. Sci..

[12] Moriyama N., Nagasawa H., Kanezashi M., Tsuru T. (2021). Improved performance of organosilica membranes for steam recovery at moderate-to-high temperatures via the use of a hydrothermally stable intermediate layer. J. Membr. Sci..

[13] Li Z., Deng Y., Wang Z., Hu J., Haw K.G., Wang G., Kawi S. (2021). A superb water permeable membrane for potential applications in CO_2_ to liquid fuel process. J. Membr. Sci..

[14] Seshimo M., Liu B., Lee H.R., Yogo K., Yamaguchi Y., Shigaki N., Mogi Y., Kita H., Nakao S. (2021). Membrane reactor for methanol synthesis using Si-rich LTA zeolite membrane. Membranes.

[15] Sakai M., Tanaka K., Matsukata M. (2022). An experimental study of a zeolite membrane reactor for reverse water gas shift. Membranes.

[16] van Leeuwen M.E. (1994). Derivation of Stockmayer potential parameters for polar fluids. Fluid Phase Equilib..

[17] Moriyama N., Takeyama A., Yamamoto T., Sawamura K., Gonoi K., Nagasawa H., Kanezashi M., Tsuru T. (2023). Steam recovery from flue gas by organosilica membranes for simultaneous harvesting of water and energy. Nat. Commun..

[18] Breck D.W. (1974). Zeolite Molecular Sieves.

[19] Aoki K., Tuan V.A., Falconer J.L., Noble R.D. (2000). Gas permeation properties of ion-exchanged ZSM-5 zeolite membranes. Micropor. Mesopor. Mater..

[20] Kusakabe K., Kuroda T., Uchino K., Hasegawa Y., Morooka S. (1999). Gas permeation properties of ion-exchanged faujasite-type zeolite membranes. AIChE J..

[21] Hasegawa Y., Watanabe K., Kusakabe K., Morooka S. (2001). The separation of CO_2_ using Y-type zeolite membranes ion-exchanged with alkali metal cations. Sep. Purif. Technol..

[22] Sakai M., Sasaki Y., Tomono T., Seshimo M., Matsukata M. (2019). Olefin selective Ag-exchanged X-type zeolite membrane for propylene/propane and ethylene/ethane separation. ACS Appl. Mater. Interfaces.

[23] Sakai M., Fujimaki N., Sasaki Y., Yasuda N., Seshimo M., Matsukata M. (2020). Preferential adsorption of propylene over propane on a Ag-exchanged X-type zeolite membrane. ACS Appl. Mater. Interfaces.

[24] Zhu M., An X., Gui T., Wu T., Li Y., Chen X. (2023). Effects of ion-exchange on the pervaporation performance and microstructure of NaY zeolite membrane. Chin. J. Chem. Eng..

[25] Sakai M., Tsuzuki Y., Fujimaki N., Matsukata M. (2021). Olefin recovery by *BEA-type zeolite membrane: Affinity-based separation with olefin–Ag^+^ interaction. Chem. Asian J..

[26] Hong M., Li S., Funke H.F., Falconer J.L., Noble R.D. (2007). Ion-exchanged SAPO-34 membranes for light gas separations. Micropor. Mesopor. Mater..

[27] Biligetu T., Wang Y., Nishitoba T., Otomo R., Park S., Mochizuki H., Kondo J.N., Tatsumi T., Yokoi T. (2017). Al distribution and catalytic performance of ZSM-5 zeolites synthesized with various alcohols. J. Catal..

[28] Watanabe R., Yokoi T., Tatsumi T. (2011). Synthesis and application of colloidal nanocrystals of the MFI-type zeolites. J. Colloid Interface Sci..

[29] Hirota Y., Betsuno R., Hotta Y., Li X., Miyake K., Nishiyama N. (2022). Catalytic performance of Zn-containing MFI zeolites in acetone-to-aromatics reactions. Micropor. Mesopor. Mater..

[30] Tang Z., Kim S.-J., Gu X., Dong J. (2009). Microwave synthesis of MFI-type zeolite membranes by seeded secondary growth without the use of organic structure directing agents. Micropor. Mesopor. Mater..

[31] Hasegawa Y., Abe C., Natsui M., Ikeda A. (2021). Gas permeation properties of high-silica CHA-type zeolite membrane. Membranes.

[32] Olson D.H., Haag W.O., Borghard W.S. (2000). Use of water as a probe of zeolitic properties: Interaction of water with HZSM-5. Micropor. Mesopor. Mater..

[33] Bolis V., Busco C., Ugliengo P. (2006). Thermodynamic study of water adsorption in high-silica zeolites. J. Phys. Chem. B.

[34] Ohlin L., Bazin P., Thibault-Starzyk F., Hedlund J., Grahn M. (2013). Adsorption of CO_2_, CH_4_, and H_2_O in zeolite ZSM-5 studied using in situ ATR-FTIR spectroscopy. J. Phys. Chem. C.

[35] Yang S., Navrotsky A. (2000). Energetics of formation and hydration of ion-exchanged zeolite Y. Micropor. Mesopor. Mater..

[36] Sakai M., Sasaki Y., Kaneko T., Matsukata M. (2021). Contribution of pore-connectivity to permeation performance of silicalite-1 membrane; Part I, Pore volume and effective pore size. Membranes.

[37] Hedlund J., Noack M., Kölsch P., Crease D., Caro J., Sterte J. (1999). ZSM-5 membranes synthesized without organic templates using a seeding technique. J. Membr. Sci..

[38] Noack M., Kölsch P., Caro J., Schneider M., Toussaint P., Sieber I. (2000). MFI membranes of different Si/Al ratios for pervaporation and steam permeation. Micropor. Mesopor. Mater..

[39] Au L.T.Y., Chau J.L.H., Ariso C.T., Yeung K.L. (2001). Preparation of supported Sil-1, TS-1 and VS-1 membranes Effects of Ti and V metal ions on the membrane synthesis and permeation properties. J. Membr. Sci..

[40] Au L.T.Y., Yeung K.L. (2001). An investigation of the relationship between microstructure and permeation properties of ZSM-5 membranes. J. Membr. Sci..

[41] Algieri C., Bernardo P., Golemme G., Barbieri G., Drioli E. (2003). Permeation properties of a thin silicalite-1 (MFI) membrane. J. Membr. Sci..

[42] Lai Z., Tsapatsis M. (2004). Gas and organic vapor permeation through *b*-oriented MFI membranes. Ind. Eng. Chem. Res..

[43] Zhao Q., Wang J., Chu N., Yin X., Yang J., Kong C., Wang A., Lu J. (2008). Preparation of high-permeance MFI membrane with the modified secondary growth method on the macroporous α-alumina tubular support. J. Membr. Sci..

[44] Xiao W., Yang J., Lu J., Wang J. (2009). Preparation and characterization of silicalite-1 membrane by counter-diffusion secondary growth. J. Membr. Sci..

[45] Xiao W., Yang J., Lu J., Wang J. (2009). A novel method to synthesize high performance silicalite-1 membrane. Sep. Purif. Technol..

[46] Zhu X., Wang H., Lin Y.S. (2020). Effect of the membrane quality on gas permeation and chemical vapor deposition modification of MFI-type zeolite membranes. Ind. Eng. Chem. Res..

[47] Xiao W., Yang J., Shen D., Lu J., Wang J. (2010). Synthesis and property of silicalite-1 membranes by restricting growth method with dilute solution. Micropor. Mesopor. Mater..

[48] Qiu L., Kumakiri I., Tanaka K., Chen X., Kita H. (2017). Effect of seed crystal size on the properties of silicalite-1 membranes synthesized in a fluoride containing medium. J. Chem. Eng. Jpn..

[49] Wang Q., Wu A., Zhong S., Wang B., Zhou R. (2017). Highly (*h0h*)-oriented silicalite-1 membranes for butane isomer separation. J. Membr. Sci..

[50] Wu A., Tang C., Zhong S., Wang B., Zhou J., Zhou R. (2019). Synthesis optimization of (*h0h*)-oriented silicalite-1 membranes for butane isomer separation. Sep. Purif. Technol..

[51] Tanizume S., Yoshimura T., Ishii K., Nomura M. (2020). Control of sequential MTO reactions through an MFI-type zeolite membrane contactor. Membranes.

[52] Ma B., Zhu Y., Hong H., Cui L., Gao H., Zhao D., Wang B., Zhou R., Xing W. (2022). Improved silicalite-1 membranes on 61-channel monolithic supports for *n*-butane/i-butane separation. Sep. Purif. Technol..

