# Supplementary material for: Gas and Steam Permeation Properties of Cation-Exchanged ZSM-5 Membrane
Source: Membranes (Basel). 2025 Mar 1;15(3):70. doi: 10.3390/membranes15030070 (PMC11943978; doi:10.3390/membranes15030070)
Supplement: Supplementary file 1 [file membranes-15-00070-s001.zip › membranes-3464332-supplementary.pdf]

Gas and Steam Permeation Properties of Cation-Exchanged ZSM-5 Membrane

Yuichiro Hirota [1,*] , Masaki Nakai [1], Kasumi Tani [1], Koya Sakane [1], Ayumi Ikeda [2], Yasuhisa Hasegawa [2] and Sadao Araki [3]

[1] Department of Life Science and Applied Chemistry, Nagoya Institute of Technology, Gokiso-cho, Showa-ku, Nagoya 466-8555, Japan
[2] National Institute of Advanced Industrial Science and Technology (AIST), Research Institute for Chemical Process Technology, 4-2-1 Nigatake, Miyagino-ku, Sendai 983-8551, Japan; a-ikeda@aist.go.jp (A.I.); yasuhisa-hasegawa@aist.go.jp (Y.H.)
[3] Department of Chemical Engineering, Kansai University, 3-35 Yamatecho 3-Chome, Suita 564-8680, Japan; araki_sa@kansai-u.ac.jp
* Correspondence: hirota.yuichiro@nitech.ac.jp

Supplementary Materials

Table of contents

1. Permeation and separation tests
2. XRD patterns of powder samples
3. FE-SEM images and EDS spectra of membranes
4. Steam adsorption isotherm
5. $p\ V^{-1}$ vs p plot
6. Langmuir constant
7. Comparison of separation performance for H_2/SF_6

1. Permeation and separation tests

Figure S1 shows a schematic diagram of the gas permeation test apparatus. Flow rates of the feed and sweep gas in the unary gas permeation test are listed in **Table S1**. The permeated gases were collected by sweeping gas. Nitrogen was used for the H_2 permeation test. And, H_2 was used for the N_2 and SF_6 permeation tests.

In the binary H_2O/H_2 permeation tests, total flow rate of the feed stream was 200 cm^3(STP)/min. To control H_2O partial pressure in the feed stream, flow rate of H_2 with and without the saturator was adjusted. Nitrogen was used as the sweep gas, and its flow rate was 300 or 400 cm^3(STP)/min.

In each permeation test, the permeated gases were analyzed using a gas chromatograph equipped with a TCD detector (GC-8A, Shimadzu, Kyoto, Japan). Molar ratio of the permeated gas and the sweep gas was determined by standard curves. The permeance, Π [mol m^{-2}s^{-1}Pa^{-1}] was calculated by the following equation

$$\Pi = u\, r\, A^{-1}\, \Delta p^{-1}$$

where u is flow rate of the sweep gas [mol s^{-1}], r is molar ratio of the permeated gas and the sweep gas [-], A is effective of membrane area, and Δp is partial pressure difference in the feed and permeated side [Pa]. In this study, detection limit is 2×10^{-11} mol m^{-2}s^{-1}Pa^{-1}.

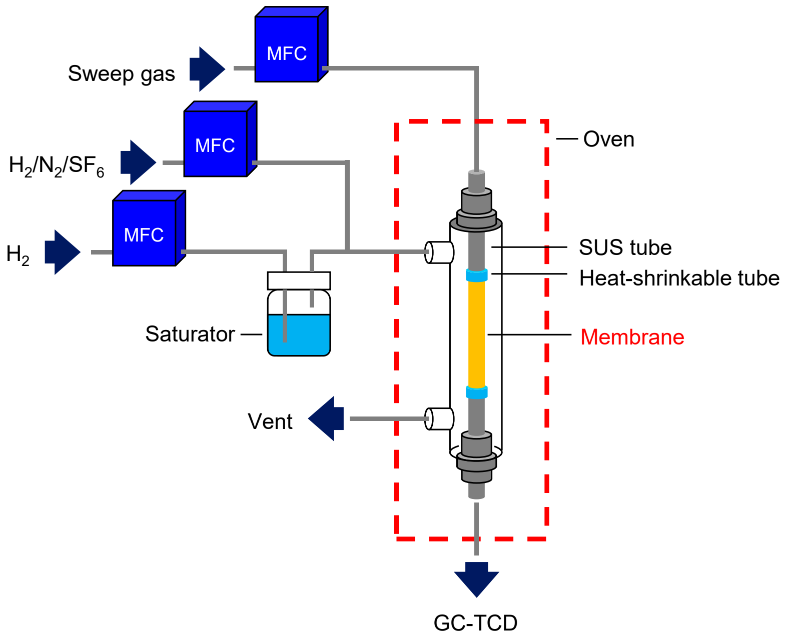

Figure S1. Schematic of the gas permeation test apparatus.

Table S1 Gas flow rate in the unary gas permeation test.

Measurement gas	Flow rate [cm^3(STP)/min]	
	Feed gas	Sweep gas
H_2	200	400
N_2	200	100
SF_6	100	20

2. XRD patterns of powder samples

Figure S2 shows XRD patterns of ZSM-5 powder samples.

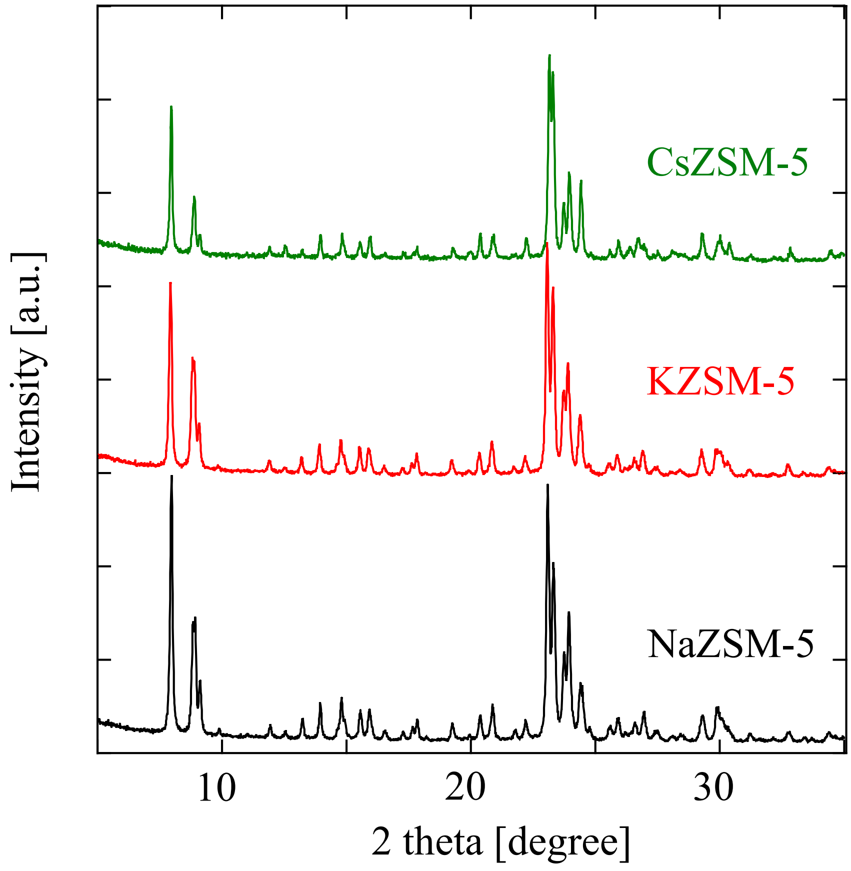

Figure S2. XRD patterns of ZSM-5 powder samples.

3. FE-SEM image and EDS spectra of membranes

Figure S3 shows cross-sectional FE-SEM image and EDS spectra of the ZSM-5 membranes.

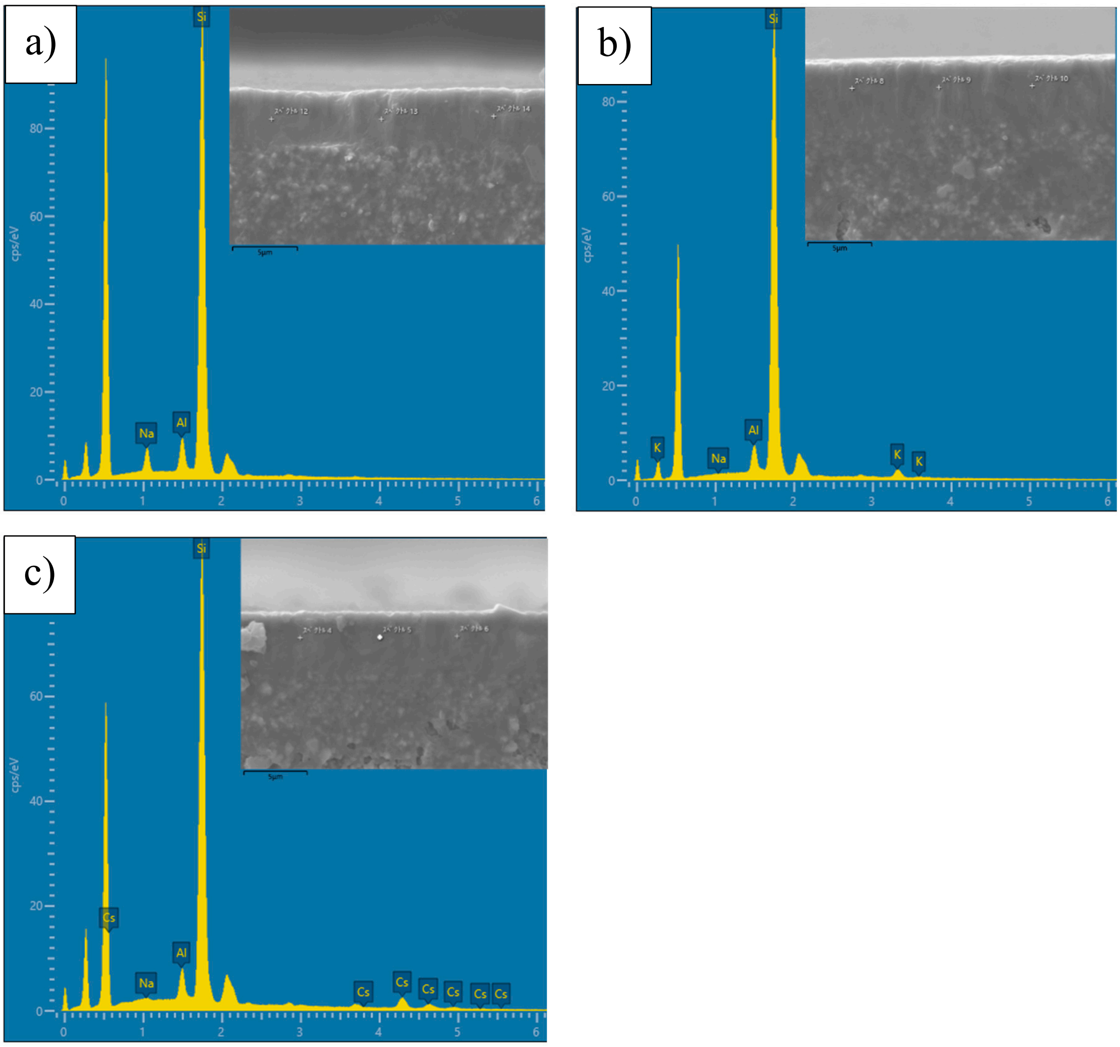

Figure S3. Cross-sectional FE-SEM images and EDS spectra of (a) NaZSM-5, (b) KZSM-5 and (c) CsZSM-5 membranes.

4. Steam adsorption isotherm

Figure S4 shows the isotherms measured at 323 and 343 K.

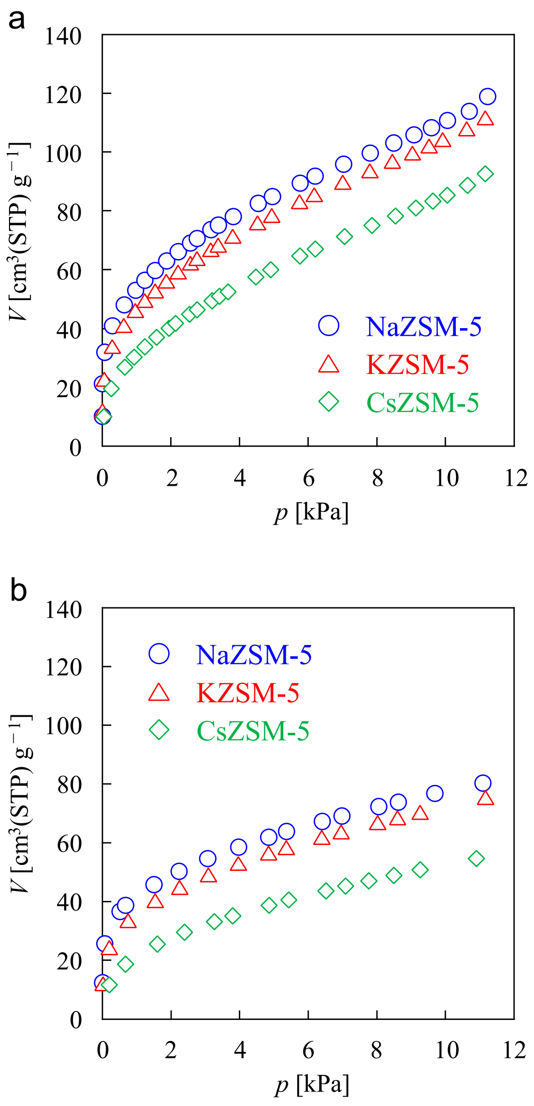

Figure S4. Steam adsorption isotherm of the ZSM-5 powder samples at (a) 323 and (b) 343 K.

5. $p\ V^{-1}$ vs p plot

Figure S5 shows the $p\ V^{-1}$ vs p plot at 323 and 343 K.

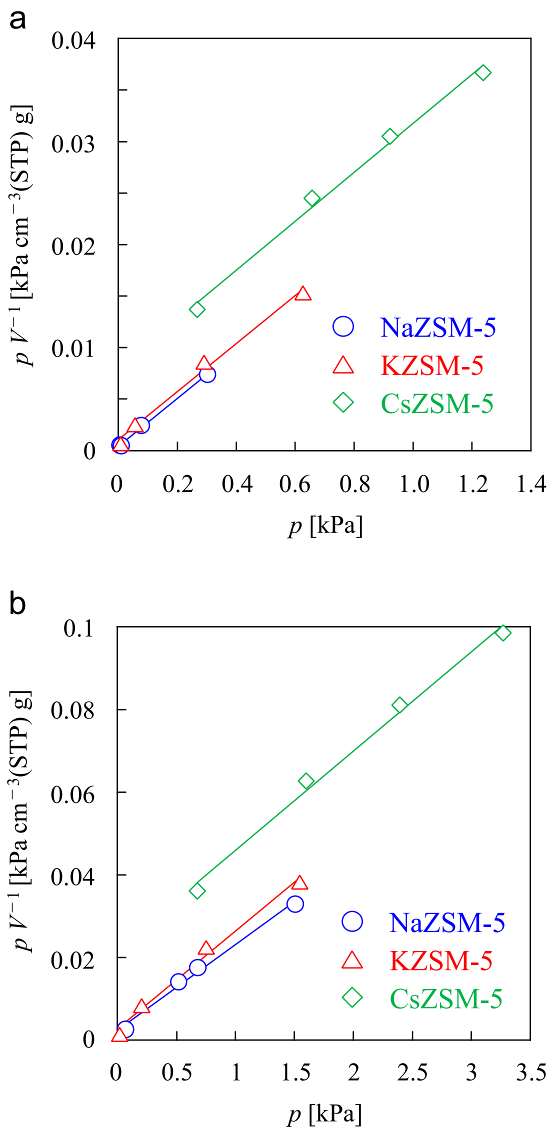

Figure S5. $p\ V^{-1}$ vs p plot of the ZSM-5 powder samples at (a) 323 and (b) 343 K.

6. Langmuir constant

Table S2 lists the calculated values of the Langmuir constant, K.

Table S2. Langmuir constants for the ZSM-5 powder samples.

Temperature [K]	K [kPa^{-1}]		
	NaZSM-5	KZSM-5	CsZSM-5
303	1.5×10^2	36	9.0
323	58	23	3.0
343	8.4	8.8	1.1

7. Comparison of separation performance for H_2/SF_6

Figure S6 shows the relationship between H_2 permeance and H_2/SF_6 permselectivity of the synthesized NaZSM-5 membranes and other MFI-type zeolite membranes [30, 37–52].

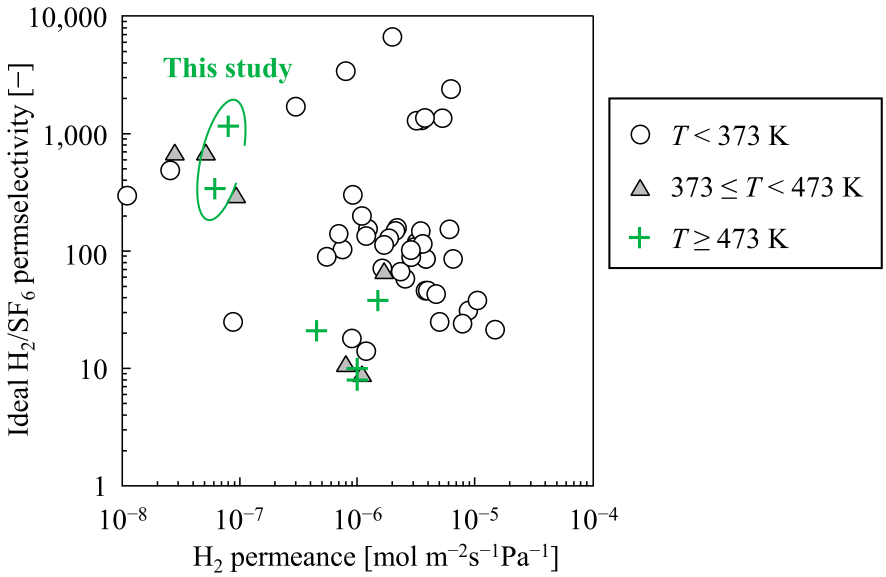

Figure S6. Relationship of H_2 permeance and H_2/SF_6 permeation ratio of MFI-type zeolite membranes.
